# Association of weight-adjusted-waist index with type 2 diabetes mellitus in Chinese urban adults: a cross-sectional study

**DOI:** 10.3389/fendo.2025.1460230

**Published:** 2025-02-10

**Authors:** Qingzheng Wu, Bing Li, Yuepeng Wang, Yue Zhang, Qian Wang, Binqi Li, Wei Jing, Jing Yang, Yiming Mu

**Affiliations:** ^1^ Department of Endocrinology, The First Medical Center of Chinese People’s Liberation Army (PLA) General Hospital, Beijing, China; ^2^ School of Medicine, Nankai University, Tianjin, China; ^3^ Department of Anesthesiology, The First Medical Center of Chinese People’s Liberation Army (PLA) General Hospital, Beijing, China; ^4^ Nanjing Drum Tower Hospital, The Affiliated Hospital of Nanjing University Medical School, Nanjing, China

**Keywords:** obesity, central adiposity, type 2 diabetes mellitus, weight-adjusted-waist index, reaction, Chinese urban adults

## Abstract

**Background:**

Recently, weight-adjusted-waist index (WWI), a new index for evaluating obesity, has been developed. This study aimed to examine the association between WWI and T2DM in Chinese urban adults.

**Method:**

A total of 5,0978 eligible participants drawn from the prospective REACTION study (Cancer Risk Assessment in Chinese People with Diabetes) were included in this study. Participants were divided into 3 groups based on baseline WWI levels. Pearson correlation analysis and binary logistic regression analysis were conducted to explore the association of WWI with T2DM risk factors and with T2DM risk.

**Results:**

The prevalence of obesity, central obesity and T2DM was 14.2%, 46.8% and 11.0% respectively, with a median age of 57 years. Logistic analysis showed that the WWI was significantly associated with the risk of T2DM. Compared to the lowest tertile of WWI (T1) serving as the reference group, the second tertile (T2) and the third tertile (T3) were associated with a 0.218-fold [1.218 (1.152, 1.288), *P <*0.001] and 0.286-fold [1.286 (1.212, 1.364), *P <*0.001] increase in the odds of developing T2DM respectively. After adjusting for all factors with the exception of the stratified variable, this association held true in age, sex, BMI, hypertension, and hyperlipidemia subgroup and was especially pronounced in those aged <60 years, BMI ≥24 kg/m^2^, and males, with interactions between WWI and age, sex, and BMI (*P* for interaction <0.05).

**Conclusion:**

WWI was positively associated with T2DM in Chinese urban adults, especially in young and middle-aged males with BMI ≥24 kg/m^2^.

## 1 Introduction

Due to the increase in obesity, sedentary lifestyle, high-energy diet, and population aging (1), the prevalence of diabetes is rising rapidly worldwide, exerting adverse with a major impact on the lives and well-being of individuals, families, and societies worldwide. By 2019, close to 0.5 billion people were living with diabetes globally, and this number is expected to increase by 25% by 2030 (2). In addition, half (50.1%) of diabetic patients are unaware of diabetes, and this part of the population has a greater risk of diabetic complications (3). Asia has been experiencing a rapidly growing T2DM epidemic, with China and India being the two major centers in Asia (4). Diabetes and its related complications (such as kidney failure, peripheral arterial disease, cardiovascular disease, and infection, etc.) have a serious effect on people’s quality of life and require a large amount of health services, which greatly aggravates the social burden.

Obesity jeopardizes public health worldwide, and has become the fifth leading cause of death worldwide (5) and the leading cause of preventable death in China (6). According to the latest estimates, nearly 14% of men and 20% of women in the world’s population (over 1 billion people in all) will be obese by 2030 (7). Obesity is now recognized as a chronic, recurrent and multifactorial disease and also as a major risk factor for non-communicable diseases (8). Obesity can cause complications through anatomical and metabolic effects, such as obstructive sleep, diabetes, cardiovascular disease (CVD), non-alcoholic fatty liver disease (NAFLD), and tumor, the multi-organ impairment and disease burden caused by these diseases may become irreversible without timely intervention (9, 10). Obesity is a major risk factor for T2DM, and weight gain and obesity are significantly associated with diabetes incidence (11). Therefore, an effective and accurate obesity assessment parameter is essential to identifying individuals at high risk of diabetes and preventing diabetes.

Body mass index (BMI) and waist circumference (WC) are the most commonly used indexes to evaluate obesity, and accumulated data have shown that they are closely related to the increased prevalence of diabetes. However, those indexes can’t fully reflect the characteristic of obesity, such as the difference between muscle mass and fat mass, between central fat and peripheral fat, and between subcutaneous fat and visceral fat (12–14). Therefore, these traditional measures may not accurately reflect the relationship between obesity and diabetes risk. Quantitative measurements of body composition can be achieved by imaging methods such as computed tomography (CT) or magnetic resonance imaging (MRI), but those methods increase exposure to ionizing radiation and are relatively limited in clinical practice (15). Park et al. (16) proposed a new obesity indicator called “weight-adjusted-waist index (WWI)” in 2018. WWI combines the advantages of WC while diluting the correlation with BMI. Therefore, WWI mainly reflects central obesity (17). Several studies have demonstrated an association between WWI and hypertension, heart failure, hyperuricemia, kidney stones, depression, and all-cause and cardiovascular mortality (16, 18–24).

Previous studies have shown a positive correlation between WWI and the prevalence of T2DM in U.S. and Japan (25–27); however, the relationship between WWI and the prevalence of T2DM in the general adult population in large-sample urban China has not been explored. Therefore, this study aims to further investigate the relationship between WWI and T2DM through a multi-center and large-sample epidemiological survey of Chinese urban adults.

## 2 Methods

### 2.1 Study population

The study population was drawn from the REACTION (Risk Evaluation of Cancers in Chinese Diabetic Individuals) study designed to research the correlation of diabetes and prediabetes with the risk of cancer in the Chinese population based on the community. In 2011-2012, 53, 639 participants from eight regional centers (Dalian, Lanzhou, Zhengzhou, Guangzhou, Guangxi, Luzhou, Shanghai, and Wuhan) participated in the study and signed the informed consent form. The subjects of the study were permanent adult residents from 3 to 5 communities randomly sampled in the city.

**Figure 1:**
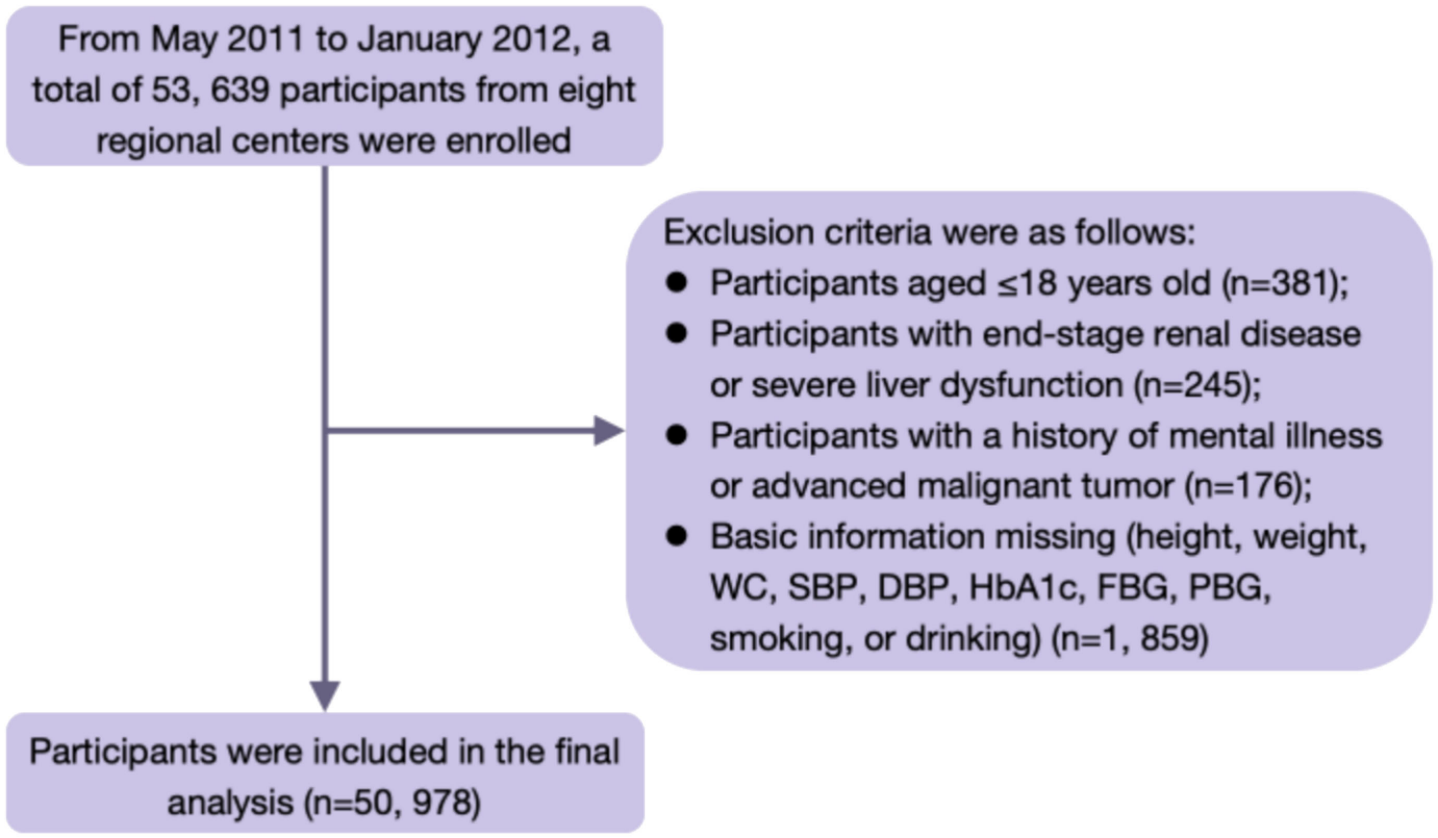
Flow chart of the study population.

Inclusion and exclusion criteria (1): Inclusion criteria: individuals aged >18 years old, without restrictions on gender proportion, having good compliance (being objective and sincere for questionnaires, and being able to accept regular follow-up) (2). Exclusion criteria: individuals diagnosed with end-stage renal disease or severe liver dysfunction (n=245); Participants with a history of mental illness or advanced malignant tumor (n=176); Participants aged ≤18 years old (n=381), lacking important data such as height, weight, WC, systolic blood pressure (SBP), diastolic blood pressure (DBP), glycated hemoglobin (HbA1c), fasting blood glucose (FBG), postprandial blood glucose (PBG), smoking status, or drinking status (n=1, 859). A total of 50, 978 participants were included (**Figure 1**).

### 2.2 Data collection

A standardized questionnaire was used by trained staff and nurses to collect basic information, medical history, medication history and lifestyle information. Height, weight and WC were measured in a standard standing position, wearing light clothing and no shoes, and recorded to 2 decimal places. WC was defined as the abdominal circumference at the lower margin of the ribs and the midpoint of the sacral line. BMI was calculated as weight divided by the square of height (kg/m^2^); waist-to-hip ratio (WHR) was calculated as waist circumference divided by hip circumference; and waist-to-height ratio (WHtR) was calculated as waist circumference divided by height. Blood pressure was measured in a standard sitting position, and patients were asked to rest for at least 5 min before measurement. Three measurements were taken every 5 minutes, and the average value was used for statistical analysis.

### 2.3 Laboratory measurements

Blood samples were collected early in the morning after fasting for at least 10 hours. Patients without diabetes underwent a 75 g glucose tolerance test, and venous blood was drawn at 0 minute and 120 minutes. Biochemical indicators included FBG, PBG, total cholesterol (TC), serum triglyceride (TG), high-density lipoprotein cholesterol (HDL-C), low-density lipoprotein cholesterol (LDL-C), alanine aminotransferase (ALT), aspartate aminotransferase (AST), fasting insulin and HbA1c, serum creatinine (CREA), etc. The estimated glomerular filtration rate (eGFR) was estimated using the CKD-EPI formula updated by lnker et al. (28) in 2021. Fasting plasma insulin levels were measured by glucose oxidase-peroxidase method. Mid-morning clean urine was collected for urinary albumin/creatinine ratio (UACR) determination.

### 2.4 Variable definition

Overweight was defined as BMI ≥24kg/m^2^, obesity was defined as BMI ≥28kg/m^2^, central obesity was defined as male: WC ≥90cm; Female: WC ≥85cm. Hypertension was defined as self-reported hypertension and/or mean systolic/diastolic blood pressure ≥140/90mmHg over three measurements. Diabetes was defined as self-reported diabetes and/or newly diagnosed diabetes (FBG ≥7.0mmol/L and/or PBG ≥11.1mmol/L and/or HbA1c ≥6.5%). Hyperlipidemia was defined as self-reported hyperlipidemia and/or TC ≥6.2mmol/L and/or TG ≥2.3 mmol/L and/or LDL-C ≥4.1mmol/L. WWI were calculated as WC divided by the square root of weight (cm/ $\sqrt{\text{kg}}$ ). All subjects were divided into three groups according to the tertiles of WWI: T1 (≤10.47), T2 (10.48-11.13), T3 (≥11.14).

### 2.5 Statistical analysis

All statistical analyses were performed using SPSS version 27.0 (IBM, Chicago, IL, USA). Study participants were divided into three groups based on the tertiles of WWI. Normally distributed continuous variables were expressed as mean ± standard deviation (SD), and non-normally distributed continuous variables were expressed as median (interquartile range). Categorical variables were expressed as frequencies and percentages. The χ2 test was used to calculate the comparison of count data between groups, with *P <*0.05 as the statistically significant difference. For measurement data normality analysis was performed firstly, and ANOVA was used for comparisons if they were met, and non-parametric tests were used if they were not. Some covariates had missing values, although the extent of missing data for each variable was less than 20%. These missing values were addressed through means of mean imputation and regression imputation. The variance inflation factor (VIF) was used to assess multicollinearity between predictor variables, with a threshold VIF value of less than 10 indicating the absence of multicollinearity, as shown in **Supplementary Table S1**. Pearson correlation analysis was used to examine the association between WWI and clinical risk factors of T2DM. Logistic regression was used to analyze the association between WWI and T2DM, and the odds ratio (OR) and 95% confidence interval (CI) were calculated. We developed three models. Model 1 was not adjusted for confounding factors. Model 2 was adjusted for age, sex, BMI, SBP, DBP, heart rate (HR), drinking status, and smoking status. Model 3 was adjusted for TG, LDL-C, HDL-C, ALT, AST, eGFR, CVD, and use of antihypertensive and lipid-lowering drugs based on model 2. In addition, we performed stratified analyses by age, sex, BMI, hypertension, and hyperlipidemia as follows: by age (<60 and ≥60 years old, representing the middle-aged and elderly population), sex (male and female), BMI (<24 kg/m^2^ and ≥24 kg/m^2^, representing normal weight or overweight/obesity), hypertension status, and hyperlipidemia status. All statistical tests were two-sided, and *P <*0.05 was considered statistically significant.

We performed several sensitivity analyses to evaluate the robustness of the results. First, considering potential biases, we excluded missing data from the dataset. In addition, considering the strong relationship between obesity and diabetes, we excluded participants with BMI ≥28kg/m^2^.

## 3 Result

### 3.1 Clinical characteristics of the study population

A total of 50,978 participants were included in the analysis. All study subjects were divided into three groups based on the tertiles of WWI. **Table 1** shows the baseline characteristics of the study population in the WWI tertiles and the comparison among the three groups with different WWI levels. The majority of the participants were women (68.6%), and most of them were middle-aged and elderly, with a median age of 57 years. Compared with participants in the T1 group, those in the T3 group were older, had higher values of SBP, DBP, BMI, WC, HC, WHR, WHtR, higher levels of TyG, HOMA-IR, FBG, PBG, HbA1c, fasting insulin, UACR, serum TC, TG, LDL-C, HDL-C, lower levels of eGFR and education, lower proportion of current smokers and alcohol drinkers (all *P <*0.001). In the total population, the prevalence of obesity, central obesity and T2DM was 14.2%, 46.9% and 11.0% respectively. With the increase of WWI tertiles, the prevalence of T2DM increased (*P*-trend <0.001). In addition, the prevalence of hypertension, hyperlipidemia, CVD and NAFLD also increased gradually with the higher WWI tertiles (*P*-trend <0.001). Moreover, participants in the T3 group had a higher proportion of medication use (antihypertensive, lipid-lowering, oral glucose-lowering and insulin) than those in the T1 group.

**Table 1:** Basic characteristics of participants by WWI tertile.

Variable	Total (n = 50,978)	WWI ≤10.47 (n = 17,003)	10.48 ≤WWI ≤11.13 (n = 16,976)	WWI ≥11.14 (n = 16,996)	*P*
Age, years	57 (51, 63)	54 (48, 59)	56 (51, 62)	61 (55, 69)	<0.001
Male, n (%)	16024 (31.4)	5951 (35)	6189 (36.4)	3884 (22.9)	<0.001
SBP, mmHg	129.33 (117.00, 143.67)	123.64 (112.33, 137.00)	129.33 (117.43, 143.00)	135.33 (122.00, 150.00)	<0.001
DBP, mmHg	76.67 (70.00, 84.00)	75.33 (68.67, 82.67)	77.33 (70.67, 84.67)	77.33 (70.67, 84.67)	<0.001
Pulse, (bpm)	78.33 (71.67, 86.33)	78 (71.33, 85.67)	78.33 (71.35, 86.33)	78.67 (72.00, 86.67)	<0.001
Height, cm	159.5 (154.3, 165.0)	161.20 (156.40, 167.00)	160.00 (155.00, 166.00)	156.56 (152.00, 162.00)	<0.001
Weight, kg	61.80 (55.00, 69.00)	60.00 (54.00, 68.00)	63.00 (56.30, 70.20)	61.90 (55.00, 69.00)	<0.001
HC, cm	96.00 (91.20, 101.00)	93.00 (89.00, 98.00)	97.00 (92.10, 101.00)	99.00 (94.00, 104.00)	<0.001
WC, cm	85.00 (79.00, 92.00)	78.00 (72.00, 83.00)	85.57 (81.00, 91.00)	92.00 (87.00, 98.00)	<0.001
BMI, kg/m^2^	24.22 (22.06, 26.50)	23.15 (21.11, 25.28)	24.50 (22.51, 26.70)	25.00 (22.81, 27.41)	<0.001
WHR	0.89 (0.84, 0.93)	0.83 (0.80, 0.87)	0.89 (0.85, 0.92)	0.93 (0.90, 0.97)	<0.001
WHtR	0.53 (0.49, 0.57)	0.48 (0.45, 0.51)	0.53 (0.51, 0.56)	0.59 (0.56, 0.62)	<0.001
FBG, mmol/L	5.54 (5.12, 6.17)	5.42 (5.04, 5.92)	5.56 (5.14, 6.20)	5.65 (5.20, 6.40)	<0.001
PBG, mmol/L	7.42 (6.02, 9.70)	6.83 (5.66, 8.57)	7.53 (6.11, 9.77)	8.01 (6.42, 10.85)	<0.001
Ins0, uU/mL	7.30 (5.30, 10.20)	6.3 (4.6, 8.8)	7.70 (5.60, 10.50)	8.10 (5.90, 11.30)	<0.001
HbA1c, (%)	5.90 (5.60, 6.30)	5.8 (5.5, 6.1)	5.90 (5.60, 6.30)	6.00 (5.60, 6.40)	<0.001
TyG	8.71 (8.33, 9.14)	8.54 (8.19, 8.95)	8.77 (8.39, 9.19)	8.83 (8.45, 9.24)	<0.001
HOMA-IR	1.86 (1.28, 2.74)	1.55 (1.09, 2.26)	1.96 (1.36, 2.84)	2.12 (1.47, 3.12)	<0.001
HDL‐C, mmol/L	1.28 (1.07, 1.51)	1.31 (1.08, 1.56)	1.25 (1.05, 1.48)	1.28 (1.08, 1.50)	<0.001
LDL‐C, mmol/L	2.91 (2.31, 3.53)	2.77 (2.19, 3.39)	2.91 (2.33, 3.52)	3.03 (2.43, 3.64)	<0.001
TC, mmol/L	5.01 (4.24, 5.77)	4.48 (4.07, 5.61)	5.01 (4.25, 5.78)	5.18 (4.41, 5.92)	<0.001
TG, mmol/L	1.33 (0.95, 1.93)	1.16 (0.84, 1.67)	1.41 (1.00, 2.04)	1.46 (1.04, 2.06)	<0.001
ALT, U/L	15.00 (11.00, 20.00)	15.00 (10.00, 18.00)	16.00 (11.00, 21.00)	15.00 (11.00, 21.00)	<0.001
AST, U/L	20.00 (16.00, 24.00)	19.00 (16.00, 23.00)	20.00 (16.00, 24.00)	20.00 (17.00, 25.00)	<0.001
GGT, U/L	20.00 (14.00, 31.00)	18.00 (13.00, 27.00)	21.00 (15.00, 33.00)	21.00 (15.00, 33.00)	<0.001
UACR, mg/g	9.17 (5.10, 18.72)	8.05 (4.44, 15.82)	9.20 (5.10, 18.53)	10.58 (5.82, 22.25)	<0.001
CREA, μmol/L	65.40 (59.10, 73.30)	65.50 (59.10, 73.60)	66.00 (59.60, 74.40)	64.70 (58.60, 72.20)	<0.001
eGFR, mL/min/1.73m²	98.57 (88.79, 105.11)	101.09 (91.74, 107.21)	99.06 (89.61, 105.12)	95.46 (85.18, 102.65)	<0.001
Education, n (%)					
Illiteracy	2,361 (4.6)	395 (2.3)	587 (3.5)	1,379 (8.1)	<0.001
Primary school	6,333 (12.4)	1,366 (8.0)	1,854 (10.9)	3,113 (18.3)	<0.001
Junior high school	16,950 (33.2)	5,168 (30.4)	5,943 (35.0)	5,839 (34.4)	<0.001
Senior high school	17,891 (35.1)	7,137 (42.0)	6,104 (35.9)	4,650 (27.4)	<0.001
College	6,830 (13.4)	2,717 (16.0)	2,285 (13.5)	1,828 (10.8)	<0.001
Smoking status, n (%)					
No	43,202 (84.7)	14,048 (82.6)	13,994 (82.4)	15,160 (89.2)	<0.001
Occasional smokers	1,223 (2.4)	468 (2.8)	466 (2.7)	289 (1.7)	<0.001
Regular smokers	5,860 (11.4)	2,241 (13.2)	2,260 (13.3)	1,319 (7.8)	<0.001
Drinking status, n (%)					
No	37,540 (73.6)	11,841 (69.6)	12,047 (70.9)	13,652 (80.3)	<0.001
Occasional drinkers	9,327 (18.3)	3,731 (21.9)	3,354 (19.8)	2,242 (13.2)	<0.001
Regular drinkers	3,336 (6.5)	1,159 (6.8)	1,310 (7.7%)	867 (5.1%)	<0.001
Prevalence of diseases, n (%)					
New-onset diabetes	5,620 (11.0)	1,214 (7.1)	1,838 (10.8)	2,568 (15.1)	<0.001
Prediabetes	5,272 (10.3)	1,582 (9.3)	1,904 (11.2)	1,786 (10.5)	<0.001
Diabetes mellitus history	7,823 (15.3)	1,908 (11.2)	2,751 (16.2)	3,164 (18.6)	<0.001
Hypertension history	11,472 (22.5)	2,509 (14.8)	3,797 (22.4)	5,166 (30.4)	<0.001
Dyslipidemia history	4,593 (9.0)	1,112 (6.5)	1,654 (9.7)	1,827 (10.7)	<0.001
CVD history	2,724 (5.3)	512 (3.0)	851 (5.0)	1,361 (8.0)	<0.001
NAFLD history	4,042 (7.9)	897 (5.3)	1,531 (9.0)	1,614 (9.5)	<0.001
Medication, n (%)					
Hypoglycemic drugs	3,998 (7.8)	821 (4.8)	1,305 (7.7)	1,872 (11.0)	<0.001
Antihypertensive drugs	7,631 (15.0)	1,608 (9.5)	2,476 (14.6)	3,547 (20.9)	<0.001
Lipid-lowering drugs	503 (1.0)	119 (0.7)	180 (1.1)	204 (1.2)	<0.001
Insulin	1,094 (2.1)	243 (1.4)	345 (2.0)	506 (3.0)	<0.001

Data are medians (interquartile ranges) for continuous variables or percentages for categorical variables.

BMI, body mass index; WHR, waist-to-hip ratio; WHtR, Waist-height ratio; WC, waist circumference; SBP, systolic blood pressure; DBP, diastolic blood pressure; FBG, fasting blood glucose; PBG, postprandial blood glucose; TC, total cholesterol; TGs: triglycerides; LDL-C, low-density lipoprotein cholesterol; HDL-C, high-density lipoprotein cholesterol; AST, aspartate aminotransferase; ALT, alanine aminotransferase; UA, uric acid; eGFR, estimated glomerular filtration rate; CREA, creatinine; UACR, urinary albumin/creatinine ratio; CVD, cardiovascular disease; NAFLD, non-alcoholic fatty liver disease.

### 3.2 Correlations of WWI with T2DM-related clinical parameters

We used Pearson correlation analysis to examine the association between WWI and T2DM-related clinical parameters. The results shows that WWI was positively correlated with age, WC, WHR, SBP, FBG, PBG, HbA1c, and Scr, and was negatively correlated with BMI, DBP, TC, TG, ALT, LDL-C, and eGFR (all *P <*0.05). No significant correlation was observed between WWI and HDL-C, AST (*P <*0.05) (**Table 2**).

**Table 2:** Correlations of WWI with T2DM-related clinical parameters.

Variable	Correlation coefficient	*P* value
Age, years	0.394	<0.001
Height, cm	-0.267	<0.001
BMI, kg/m^2^	-0.083	<0.001
WC, cm	0.672	<0.001
WHR	0.083	<0.001
SBP, mmHg	0.114	<0.001
DBP, mmHg	-0.066	<0.001
FBG, mmol/L	0.186	<0.001
PBG, mmol/L	0.203	<0.001
HbA1c, (%)	0.163	<0.001
TC, mmol/L	-0.067	<0.001
TGs, mmol/L	-0.042	0.001
HDL-C, mmol/L	0.017	0.189
LDL-C, mmol/L	-0.046	<0.001
ALT, U/L	-0.074	<0.001
AST, U/L	-0.019	0.146
CREA, μmol/L	0.248	<0.001
eGFR, mL/min/1.73m^2^	-0.317	<0.001

Pearson correlation coefficient.

BMI, body mass index; WC, waist circumference; WHR, waist-to-hip ratio; SBP, systolic blood pressure; DBP, diastolic blood pressure; FBG, fasting blood glucose; PBG, postprandial blood glucose; TC, total cholesterol; TGs, triglycerides; LDL-C, low-density lipoprotein cholesterol; HDL -C, high-density lipoprotein cholesterol; AST, aspartate aminotransferase; ALT, alanine aminotransferase; UA, uric acid; CREA, creatinine; eGFR, estimated glomerular filtration rate.

### 3.3 Association between WWI Tertile and T2DM risk in Chinese urban adults

**Table 3** shows that the risk of T2DM increased with increasing WWI tertiles (*P*-trend <0.001). In Model 1, univariate analysis showed that WWI was associated with an increased risk of T2DM. In Model2, age, sex, BMI, SBP, DBP, HR, drinking status, and smoking status were adjusted. Then, TC, TG, LDL-C, HDL, ALT, AST, eGFR, CVD, antihypertensive drugs, and lipid-lowering drugs were added to model 3. The results were shown in **Table 3**. After fully adjusting for confounding factors, WWI remained independently associated with T2DM. Compared with the lowest tertile of WWI, T2 and T3 were associated with a 0.218-fold [1.218 (1.152, 1.288), *P <*0.001] and 0.286-fold [1.286 (1.212, 1.364), *P <*0.001] increase in the odds of developing T2DM, respectively. These results underscored a significant and positive association between WWI and the risk of T2DM. Additionally, our results showed that WWI was still positively correlated with T2DM when it was a continuous variable.

**Table 3:** Association between WWI Tertile and T2DM in Chinese urban adults.

	Model 1		Model 2		Model 3	
	OR (95%CI)	*P*-trend	OR (95% CI)	*P*-trend	OR (95% CI)	*P-*trend
WWI Tertile		<0.001		<0.001		<0.001
T1	1.00		1.00		1.00	
T2	1.647 (1.564, 1.734)*		1.319 (1.249, 1.393)*		1.218 (1.152, 1.288)*	
T3	2.263 (2.152, 2.379)*		1.391 (1.313, 1.473)*		1.286 (1.212, 1.364)*	
WWI Continuous	1.427 (1.394, 1.460)	<0.001	1.145 (1.115, 1.175)*	<0.001	1.101 (1.072, 1.131)*	<0.001

*Significant data.

Model 1: unadjusted;

Model 2: adjusted for sex, age, education, SBP, SDP, HR, BMI, smoking status, drinking status;

Model 3: additionally adjusted for HDL-C, LDL-C, TG, ALT, AST, eGFR, CVD, antihypertensive drugs, lipid-lowering drugs.

### 3.4 Subgroup and sensitivity analysis

**Figure 2:**
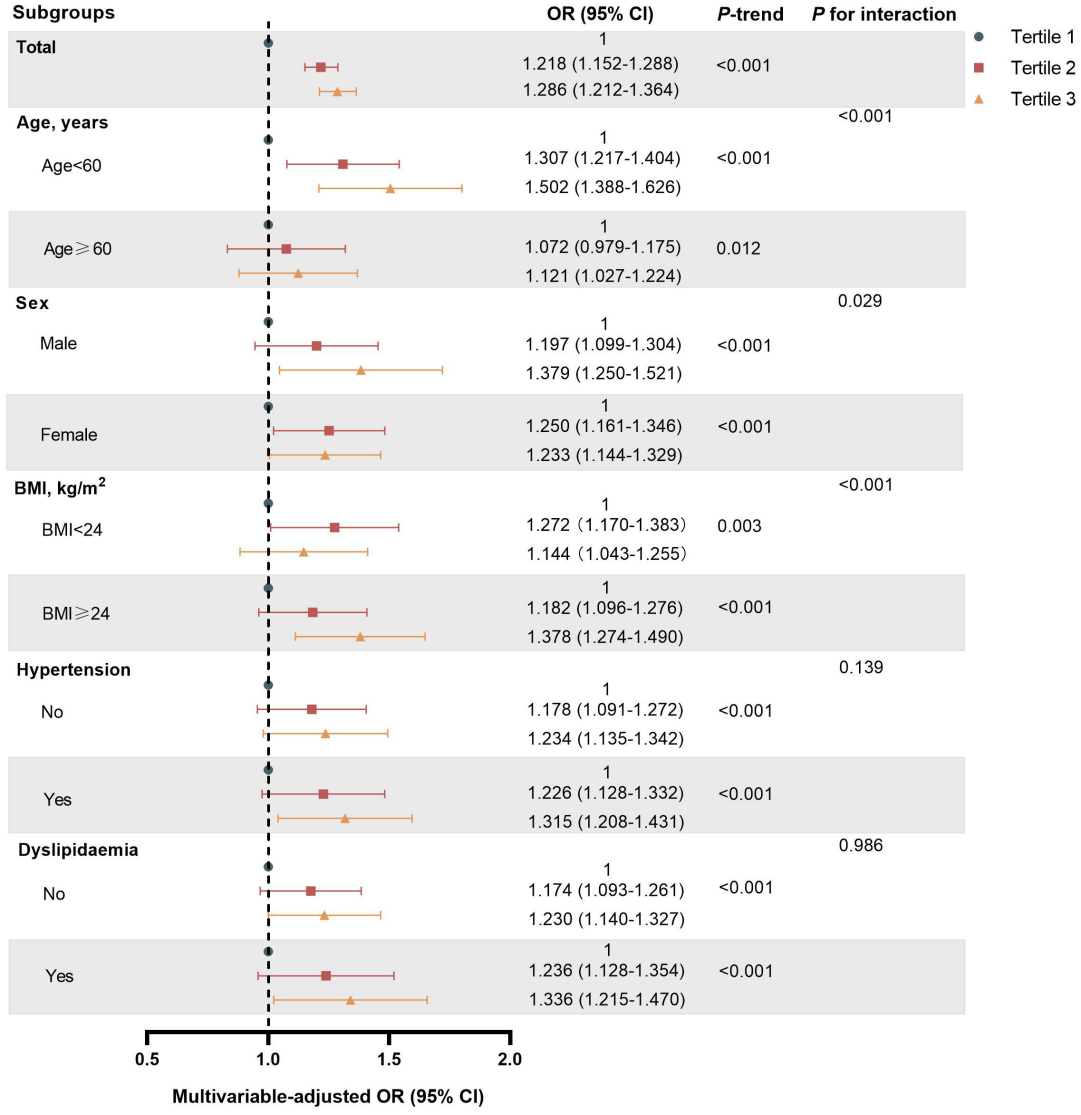
Subgroup analyses of association between WWI Tertile and T2DM risk in Chinese urban adults.

Stratified analyses were conducted based on age, sex, BMI, hypertension, and hyperlipidemia to further investigate the association between WWI and T2DM (**Figure 2**). The results showed that the ORs for T2DM significant increased with higher WWI. Of note, there was a significant interaction between WWI and age, BMI and sex (*P* for interaction <0.05). In those aged <60 years, BMI >24 kg/m^2^ and males, there was a stronger correlation between WWI and T2DM. However, the interaction with other stratification factors such as hypertension and hyperlipidemia were not significant (**Supplementary Table S2**). The results obtained after excluding missing data from the dataset are consistent with the results of the primary analysis (**Supplementary Table S3**). Furthermore, the results remained stable after the exclusion of participants BMI ≥28kg/m^2^ (**Supplementary Table S4**).

## 4 Discussion

WWI is a new anthropometric indicator to assess adiposity. In this cross-sectional study examining the relationship between WWI and T2DM among Chinese urban adults, we demonstrated a positive association between WWI and the risk of T2DM, which was consistent and stable after adjustment for a wide range of biochemical and lifestyle risk factors. Further stratification analysis revealed that the positive association between WWI and T2DM held true in age, sex, BMI, hypertension, and hyperlipidemia subgroup and was especially stronger in those aged <60 years, BMI ≥24 kg/m^2^ and males.

A cross-sectional study in U.S. adults (n = 31,001) by Zheng et al. (25) observed a strong positive correlation between WWI and the odds of T2DM, and found higher odds of T2DM among younger and non-hypertensive populations. In a prospective cohort study (n = 9,205) in the rural areas of northeast China, Yu et al. (26) found that increasing WWI was significantly associated with a higher incidence of newly diagnosed T2DM, indicating that WWI could be used as a simple and effective predictors of T2DM.Another study (27) reported that there was a positive linear association between baseline WWI and newly diagnosed T2DM in Japanese adults. Our findings were generally consistent with the above studies and further demonstrated that WWI could be a simple and effective predictor for T2DM. Moreover, consistent with previous study of Zheng et al., we also found a significant interaction between WWI and age, and showed that the association between WWI and T2DM was stronger in those aged <60 years. Likewise, other obesity index, such as WC, was demonstrated to be more associated with T2DM in individuals aged <60 years than in those aged ≥ 60 years (29). The reason may be as follows. Firstly, age affects body composition. Younger people tend to have more muscle mass, but muscle mass gradually decreases while fat content increases with age progression (30, 31). Secondly, age affects fat distribution. Studies have reported that central obesity is more significant in the elderly than in the young (32, 33).

As mentioned earlier, obesity leads to the occurrence of diabetes, but most of the previous studies were conducted using BMI and WC as indicators of obesity. However, both of BMI and WC have some disadvantages in evaluating obesity. For example, BMI neither reflects the distribution of body fat nor can differentiate between fat and muscle. Although WC can assess the degree of abdominal obesity, it could not differentiate between subcutaneous and visceral obesity. Recent studies have shown that central obesity is more closely associated with T2DM (34–36). Since WWI is derived from weight-standardized WC which mainly reflects pure central obesity independent of weight, it can be used as a better indicator of central obesity than WC. In addition, several studies have shown that WWI is positively correlated with fat mass and negatively correlated with muscle mass measured by various methods (37, 38). Thus, WWI could reflect body composition and fat distribution, and may be superior to BMI and WC in accurately evaluating central obesity.

As a better indicator of central obesity, WWI may induce diabetes through the following mechanisms: Firstly, altered adipose tissue metabolism: insulin-resistant factors such as free fatty acids (FFA), cortisol, and testosterone are active in abdominally obese individuals. FFA increases the generation of hepatic lipid and lipoprotein, and may reduce the clearance of the liver on insulin, causing insulin resistance in muscle and liver (39). Cortisol and testosterone have a “licensing” effect on adipolysis, thus amplifying the lipolytic stimulus and inducing insulin resistance in muscle (40). Secondly, altered endocrine function of adipose tissue: When adipocyte dysfunction occurs as a result of adipose tissue dilatation, there is an imbalance between pro-inflammatory and anti-inflammatory adipokines secreted by adipose tissue. For example, adipose tissue releases a variety of pro-inflammatory molecules. Aldosterone, a hormone traditionally associated with blood pressure regulation, has been implicated in the inflammatory response within adipose tissue (41). Studies have shown that aldosterone treatment of cultured adipocytes increases the expression of interleukin-6 (IL-6), plasminogen activator inhibitor-1, chemerin, and leptin, which are key players in the inflammatory cascade that can lead to insulin resistance (42). Additionally, the chronic low-grade inflammation in obesity, characterized by an increase in pro-inflammatory cytokines such as IL-6) and tumor necrosis factor-α, along with C-reactive protein, creates a state that may precipitate insulin resistance (43, 44). In contrast, anti-inflammatory factors such as adiponectin can regulate insulin sensitivity by reducing ectopic fat deposition, but high accumulation of visceral fat can reduce the concentration of adiponectin. For instance, excess aldosterone will lead to a decrease in blood adiponectin levels and correspondingly reduce its expression in visceral adipose tissue, thereby increasing susceptibility to a range of metabolic complications (45, 46). The dysregulation of the above pro-inflammatory and anti-inflammatory adipokines may have local or systemic effects on the inflammatory response, leading to the occurrence and progression of obesity-induced diabetes and other complications (47). Thus, our study further demonstrated the significance of early screening of central obesity for the prevention of diabetes and the potential of WWI as an accurate indicator to reflect central obesity.

A growing number of studies have explored the relationship between WWI and other diseases. Park et al. (16) research has found a positive linear association between WWI and cardiometabolic morbidity and mortality. In addition, two studies have showed that elevated WWI levels were independently associated with increased risks of cardiovascular and all-cause mortality in the Chinese population (19, 23). Subsequently, Fang et al. (48) found that higher levels of WWI were significantly associated with an increased risk of CVD in U.S. adults. Zhang et al. (18) found a significant correlation between WWI and risk of heart failure (HF) in a cross-sectional study, which can be used as an independent linear indicator of the risk of prevalent HF in the general population. What’ more, Li et al. (22) and Liu et al. (49) showed that WWI was positively associated with an increased risk of depression. To sum up, increased WWI levels are significantly associated with a variety of diseases and can be used for early disease screening.

The strengths of our study are the large sample size and the fact that the participants came from eight urban regions in different geographic areas of China. Nevertheless, this study had some limitations: First, some of the diseases identified in the survey were self-reported by participants, which may be underreported or overreported despite rigorous questionnaire survey according to the diagnostic criteria of the disease. Second, although we have adjusted for many important covariates, we cannot rule out any potential residual confounding. Third, since this was a cross-sectional study, causal relationship cannot be inferred, which needs to be further verified in a larger follow-up study.

## 5 Conclusion

In conclusion, this study showed that WWI was significantly and positively associated with the risk of T2DM in Chinese urban adults for the first time, and the association was especially pronounced in those aged <60 years, BMI ≥24 kg/m^2^ and males. We expect that larger prospective cohort studies will be conducted to further investigate the association between WWI and T2DM and verify the significance of WWI in screening people at high risk for T2DM.

## Data Availability

The data analyzed in this study is subject to the following licenses/restrictions: Firstly, the dataset is restricted to specific research or analytical purposes only. Secondly, the dataset is subject to copyright protection and must be used in compliance with the stated copyright notice. Finally, specific licenses or permissions may be required to access and use the dataset. Requests to access these datasets should be directed to Qingzheng Wu, wqz_own@163.com.

## References

[1] Vijan S. Type 2 diabetes. Ann Intern Med. (2019) 171:ITC65–80. doi: 10.7326/AITC201911050 31683294

[2] Saeedi P Petersohn I Salpea P Malanda B Karuranga S Unwin N. Global and regional diabetes prevalence estimates for 2019 and projections for 2030 and 2045: Results from the International Diabetes Federation Diabetes Atlas, 9th edition. Diabetes Res Clin Pract. (2019) 157:107843. doi: 10.1016/j.diabres.2019.107843 31518657

[3] Beagley J Guariguata L Weil C Motala AA. Global estimates of undiagnosed diabetes in adults. Diabetes Res Clin Pract. (2014) 103:150–60. doi: 10.1016/j.diabres.2013.11.001 24300018

[4] Home. IDF diabetes atlas. Available online at: https://diabetesatlas.org/ (Accessed April 20, 2024).

[5] Giordano A Cinti F Canese R Carpinelli G Colleluori G Di Vincenzo A. The adipose organ is a unitary structure in mice and humans. Biomedicines. (2022) 10:2275. doi: 10.3390/biomedicines10092275 36140375 PMC9496043

[6] Peng W Jian W Li T Malowany M Tang X Huang M. Disparities of obesity and non-communicable disease burden between the Tibetan Plateau and developed megacities in China. Front Public Health. (2023) 10:1070918. doi: 10.3389/fpubh.2022.1070918 36703857 PMC9873242

[7] World Obesity Federation. World Obesity Atlas 2022 [updated]. London, United Kingdom: World Obesity Federation (2022).

[8] Tsai AG Bessesen DH. Obesity. Ann Intern Med. (2019) 170:ITC33–48. doi: 10.7326/AITC201903050 30831593

[9] Ansari S Haboubi H Haboubi N. Adult obesity complications: challenges and clinical impact. Ther Adv Endocrinol Metab. (2020) 11:2042018820934955. doi: 10.1177/2042018820934955 32612803 PMC7309384

[10] Cai X Song S Hu J Zhu Q Yang W Hong J. Body roundness index improves the predictive value of cardiovascular disease risk in hypertensive patients with obstructive sleep apnea: a cohort study. Clin Exp Hypertension. (2023) 45:2259132. doi: 10.1080/10641963.2023.2259132 37805984

[11] Mokdad AH Ford ES Bowman BA Dietz WH Vinicor F Bales VS. Prevalence of obesity, diabetes, and obesity-related health risk factors, 2001. JAMA. (2003) 289:76–9. doi: 10.1001/jama.289.1.76 12503980

[12] WHO Expert Consultation. Appropriate body-mass index for Asian populations and its implications for policy and intervention strategies. Lancet. (2004) 363:157–63. doi: 10.1016/S0140-6736(03)15268-3 14726171

[13] Flegal KM Kit BK Orpana H Graubard BI. Association of all-cause mortality with overweight and obesity using standard body mass index categories. JAMA. (2013) 309:71–82. doi: 10.1001/jama.2012.113905 23280227 PMC4855514

[14] Romero-Corral A Somers VK Sierra-Johnson J Thomas RJ Collazo-Clavell ML Korinek J. Accuracy of body mass index to diagnose obesity in the US adult population. Int J Obes (Lond). (2008) 32:959–66. doi: 10.1038/ijo.2008.11 PMC2877506 18283284

[15] Garg K Chang S Scherzinger A. Obesity and diabetes: newer concepts in imaging. Obes And Diabetes. (2013) 15(5):351–62. doi: 10.1089/dia.2013.0039 23634670

[16] Park Y Kim NH Kwon TY Kim SG. A novel adiposity index as an integrated predictor of cardiometabolic disease morbidity and mortality. Sci Rep. (2018) 8:16753. doi: 10.1038/s41598-018-35073-4 30425288 PMC6233180

[17] Qin Z Chang K Yang Q Yu Q Liao R Su B. The association between weight-adjusted-waist index and increased urinary albumin excretion in adults: A population-based study. Front Nutr. (2022) 9:941926. doi: 10.3389/fnut.2022.941926 36034904 PMC9412203

[18] Zhang D Shi W Ding Z Park J Wu S Zhang J. Association between weight-adjusted-waist index and heart failure: Results from National Health and Nutrition Examination Survey 1999-2018. Front Cardiovasc Med. (2022) 9:1069146. doi: 10.3389/fcvm.2022.1069146 36588556 PMC9794568

[19] Ding C Shi Y Li J Li M Hu L Rao J. Association of weight-adjusted-waist index with all-cause and cardiovascular mortality in China: A prospective cohort study. Nutrition Metab Cardiovasc Dis. (2022) 32:1210–7. doi: 10.1016/j.numecd.2022.01.033 35277327

[20] Ding Y Xu Z Zhou X Luo Y Xie R Li Y. Association between weight-adjusted-waist index and the risk of hyperuricemia in adults: a population-based investigation. Front Endocrinol (Lausanne). (2023) 14:1236401. doi: 10.3389/fendo.2023.1236401 37900143 PMC10600370

[21] Lin W Ye Q Lin M-E. Relationship between the weight-adjusted-waist index and kidney stone: a population-based study. World J Urol. (2023) 41:3141–7. doi: 10.1007/s00345-023-04620-8 37783845

[22] Li M Yu X Zhang W Yin J Zhang L Luo G. The association between weight-adjusted-waist index and depression: Results from NHANES 2005-2018. J Affect Disord. (2024) 347:299–305. doi: 10.1016/j.jad.2023.11.073 38000467

[23] Cai S Zhou L Zhang Y Cheng B Zhang A Sun J. Association of the weight-adjusted-waist index with risk of all-cause mortality: A 10-year follow-up study. Front Nutr. (2022) 9:894686. doi: 10.3389/fnut.2022.894686 35694172 PMC9174751

[24] Zhao J Cai X Hu J Song S Zhu Q Shen D. J-shaped relationship between weight-adjusted-waist index and cardiovascular disease risk in hypertensive patients with obstructive sleep apnea: A cohort study. Diabetes Metab Syndr Obes. (2024) 17:2671–81. doi: 10.2147/DMSO.S469376 PMC11228610 38978818

[25] Zheng D Zhao S Luo D Lu F Ruan Z Dong X. Association between the weight-adjusted waist index and the odds of type 2 diabetes mellitus in United States adults: a cross-sectional study. Front Endocrinol (Lausanne). (2024) 14:1325454. doi: 10.3389/fendo.2023.1325454 38292766 PMC10824908

[26] Yu S Wang B Guo X Li G Yang H Sun Y. Weight-adjusted-waist index predicts newly diagnosed diabetes in chinese rural adults. J Clin Med. (2023) 12:1620. doi: 10.3390/jcm12041620 36836156 PMC9961347

[27] Sun H Li Y Shi J Li K Zhao Y Shang L. Weight-adjusted waist index is not superior to conventional anthropometric indices for predicting type 2 diabetes: a secondary analysis of a retrospective cohort study. Fam Pract. (2023) 40:782–8. doi: 10.1093/fampra/cmad047 37067789

[28] Inker LA Enenanya ND Coresh J Tighiouart H Wang D Sang Y. New creatinine- and cystatin C-based equations to estimate GFR without race. N Engl J Med. (2021) 385:1737–49. doi: 10.1056/NEJMoa2102953 PMC8822996 34554658

[29] Xi L Yang X Wang R Ku C Wu B Dai M. Waist circumference-years construct analysis and the incidence of type 2 diabetes: China health and nutrition survey, 1997-2015. Nutrients. (2022) 14:4654. doi: 10.3390/nu14214654 36364916 PMC9654573

[30] Kyle UG Genton L Hans D Karsegard L Slosman DO Richard C. Age-related differences in fat-free mass, skeletal muscle, body cell mass and fat mass between 18 and 94 years. Eur J Clin Nutr. (2001) 55:663–72. doi: 10.1038/sj.ejcn.1601198 11477465

[31] Gurunathan U Myles PS. Limitations of body mass index as an obesity measure of perioperative risk. Br J Anaesthesia. (2016) 116:319–21. doi: 10.1093/bja/aev541 26865129

[32] Shen W Punyanitya M Silva AM Chen J Gallagher D Sardinha LB. Sexual dimorphism of adipose tissue distribution across the lifespan: a cross-sectional whole-body magnetic resonance imaging study. Nutr Metab (Lond). (2009) 6:17. doi: 10.1186/1743-7075-6-17 19371437 PMC2678136

[33] Kuk JL Saunders TJ Davidson LE Ross R. Age-related changes in total and regional fat distribution. Ageing Res Rev. (2009) 8:339–48. doi: 10.1016/j.arr.2009.06.001 19576300

[34] Janiszewski PM Janssen I Ross R. Does waist circumference predict diabetes and cardiovascular disease beyond commonly evaluated cardiometabolic risk factors? Diabetes Care. (2007) 30:3105–9. doi: 10.2337/dc07-0945 17712026

[35] Langenberg C Sharp SJ Schulze MB Rolandsson O Overvad K Forouhi NG. Long-term risk of incident type 2 diabetes and measures of overall and regional obesity: the EPIC-InterAct case-cohort study. PloS Med. (2012) 9:e1001230. doi: 10.1371/journal.pmed.1001230 22679397 PMC3367997

[36] Decoda Study Group Nyamdorj R Qiao Q Lam TH Tuomilehto J Ho SY. BMI compared with central obesity indicators in relation to diabetes and hypertension in Asians. Obes (Silver Spring). (2008) 16:1622–35. doi: 10.1038/oby.2008.73 18421260

[37] Kim NH Park Y Kim NH Kim SG. Weight-adjusted waist index reflects fat and muscle mass in the opposite direction in older adults. Age Ageing. (2021) 50:780–6. doi: 10.1093/ageing/afaa208 33035293

[38] Kim JY Choi J Vella CA Criqui MH Allison MA Kim NH. Associations between weight-adjusted waist index and abdominal fat and muscle mass: multi-ethnic study of atherosclerosis. Diabetes Metab J. (2022) 46:747–55. doi: 10.4093/dmj.2021.0294 PMC9532169 35350091

[39] Després J-P Lemieux I. Abdominal obesity and metabolic syndrome. Nature. (2006) 444:881–7. doi: 10.1038/nature05488 17167477

[40] Björntorp P. Metabolic implications of body fat distribution. Diabetes Care. (1991) 14:1132–43. doi: 10.2337/diacare.14.12.1132 1773700

[41] Song S Cai X Hu J Zhu Q Shen D Ma H. Plasma aldosterone concentrations elevation in hypertensive patients: the dual impact on hyperuricemia and gout. Front Endocrinol (Lausanne). (2024) 15:1424207. doi: 10.3389/fendo.2024.1424207 39140032 PMC11319118

[42] Gilbert KC Brown NJ. Aldosterone and inflammation. Curr Opin Endocrinol Diabetes Obes. (2010) 17:199–204. doi: 10.1097/MED.0b013e3283391989 20422780 PMC4079531

[43] Huby A-C Antonova G Groenendyk J Gomez-Sanchez CE Bollag WB Filosa JA. Adipocyte-derived hormone leptin is a direct regulator of aldosterone secretion, which promotes endothelial dysfunction and cardiac fibrosis. Circulation. (2015) 132:2134–45. doi: 10.1161/CIRCULATIONAHA.115.018226 26362633

[44] Yudkin JS Stehouwer CD Emeis JJ Coppack SW. C-reactive protein in healthy subjects: associations with obesity, insulin resistance, and endothelial dysfunction: a potential role for cytokines originating from adipose tissue? Arterioscler Thromb Vasc Biol. (1999) 19:972–8. doi: 10.1161/01.ATV.19.4.972 10195925

[45] Côté M Bergeron J Alméras N Tremblay A Lemieux I Després JP. Adiponectinemia in visceral obesity: impact on glucose tolerance and plasma lipoprotein and lipid levels in men. J Clin Endocrinol Metab. (2005) 90:1434–9. doi: 10.1210/jc.2004-1711 15598678

[46] Shen D Cai X Hu J Song S Zhu Q Ma H. Associating plasma aldosterone concentration with the prevalence of MAFLD in hypertensive patients: insights from a large-scale cross-sectional study. Front Endocrinol (Lausanne). (2024) 15:1451383. doi: 10.3389/fendo.2024.1451383 39363897 PMC11446807

[47] Ouchi N Parker JL Lugus JJ Walsh K. Adipokines in inflammation and metabolic disease. Nat Rev Immunol. (2011) 11:85–97. doi: 10.1038/nri2921 21252989 PMC3518031

[48] Fang H Xie F Li K Li M Wu Y. Association between weight-adjusted-waist index and risk of cardiovascular diseases in United States adults: a cross-sectional study. BMC Cardiovasc Disord. (2023) 23:435. doi: 10.1186/s12872-023-03452-z 37658325 PMC10474739

[49] Liu H Zhi J Zhang C Huang S Ma Y Luo D. Association between Weight-Adjusted Waist Index and depressive symptoms: A nationally representative cross-sectional study from NHANES 2005 to 2018. J Affect Disord. (2024) 350:49–57. doi: 10.1016/j.jad.2024.01.104 38220117

