# Supplementary material for: Association of weight-adjusted-waist index with type 2 diabetes mellitus in Chinese urban adults: a cross-sectional study
Source: Front Endocrinol (Lausanne). 2025 Feb 10;16:1460230. doi: 10.3389/fendo.2025.1460230 (PMC11847671; doi:10.3389/fendo.2025.1460230)
Supplement: Supplementary file 1 [file Table1.docx]

**Supplementary Tables**

**Table S1.** Collinearity diagnostics.

| Variable | Tolerance | VIF |
| --- | --- | --- |
| WWI | 0.806 | 1.241 |
| sex | 0.576 | 1.735 |
| age | 0.470 | 2.128 |
| culutrue | 0.891 | 1.123 |
| SBP | 0.468 | 2.136 |
| SDP | 0.514 | 1.946 |
| HR | 0.921 | 1.086 |
| BMI | 0.826 | 1.211 |
| HDL-C | 0.350 | 2.858 |
| LDL-C | 0.098 | 10.228 |
| TC | 0.065 | 15.401 |
| TG | 0.353 | 2.836 |
| ALT | 0.409 | 2.446 |
| AST | 0.407 | 2.457 |
| eGFR | 0.593 | 1.687 |
| CVD | 0.928 | 1.077 |
| antihypertensive drugs | 0.847 | 1.181 |
| lipid-lowering drugs | 0.985 | 1.015 |
| smoking status | 0.679 | 1.472 |
| drinking status | 0.751 | 1.331 |

VIF = 1/(1-R2). Calculate the VIF of each variable. If the maximum VIF value≥10, remove the variable with the maximum VIF value.

Abbreviations: VIF, variance inflation factor. Other abbreviations as presented in Table 1.

**Table S2.** Subgroup analysis of the association between WWI and T2DM

|  | WWI Tertile | | | *P*-trend | *P* for Interaction |
| --- | --- | --- | --- | --- | --- |
|  | T1 (≤10.47) | T2 (10.48~11.13) | T3 (≥11.14) |  |  |
| **Total** |  |  |  |  |  |
| No. of patients | 17,003 | 16,979 | 16,996 |  |  |
| No. of cases (%) | 3,122 (18.3) | 4,589 (27.0) | 5,732 (33.7) |  |  |
| OR (95% CI) | 1.00 | 1.647 (1.564, 1.734)* | 2.263 (2.152, 2.379)* | <0.001 |  |
| Multivariable-adjusted OR (95% CI) | 1.00 | 1.218 (1.152, 1.288)* | 1.286 (1.212, 1.364)* | <0.001 |  |
| **Age, years** |  |  |  |  | <0.001 |
| Age <60 |  |  |  |  |  |
| No. of patients | 13,032 | 10,906 | 7,457 |  |  |
| No. of cases (%) | 1,912 (14.6) | 2,438 (22.3) | 1,909 (25.6) |  |  |
| OR (95% CI) | 1.00 | 1.674 (1.567, 1.789)* | 2.001 (1.864, 2.149)* | <0.001 |  |
| Multivariable-adjusted OR (95% CI) | 1.00 | 1.307 (1.217, 1.404)* | 1.502 (1.388, 1.626)* | <0.001 |  |
| Age ≥60 |  |  |  |  |  |
| No. of patients | 3,971 | 6,073 | 9,539 |  |  |
| No. of cases (%) | 1,210 (30.4) | 2,151 (30.4) | 3,823 (40.0) |  |  |
| OR (95% CI) | 1.00 | 1.251 (1.149, 1.363)* | 1.526 (1.410, 1.652)* | <0.001 |  |
| Multivariable-adjusted OR (95% CI) | 1.00 | 1.072 (0.979, 1.175) | 1.121 (1.027, 1.224)* | 0.012 |  |
| **Sex** |  |  |  |  | 0.029 |
| Male |  |  |  |  |  |
| No. of patients | 5,951 | 6,189 | 3,884 |  |  |
| No. of cases (%) | 1,435 (24.1) | 1,990 (32.1) | 1,513 (38.9) |  |  |
| OR (95% CI) | 1.00 | 1.491 (1.377, 1.615)* | 2.008 (1.840, 2.192)* | <0.001 |  |
| Multivariable-adjusted OR (95% CI) | 1.00 | 1.197 (1.099, 1.304)* | 1.379 (1.250, 1.521)* | <0.001 |  |
| Female |  |  |  |  |  |
| No. of patients | 11,052 | 10,790 | 13,112 |  |  |
| No. of cases (%) | 1,687 (15.2) | 2,599 (24.0) | 4,219 (32.1) |  |  |
| OR (95% CI) | 1.00 | 1.761 (1.645, 1.886)* | 2.634 (2.472, 2.806)* | <0.001 |  |
| Multivariable-adjusted OR (95% CI) | 1.00 | 1.250 (1.161, 1.346)* | 1.233 (1.144, 1.329)* | <0.001 |  |
| **BMI, kg/m^2^** |  |  |  |  | <0.001 |
| BMI <24 |  |  |  |  |  |
| No. of patients | 10,344 | 7,377 | 6,414 |  |  |
| No. of cases (%) | 1,531 (14.8) | 1,623 (22.0) | 1,597 (24.8) |  |  |
| OR (95% CI) | 1.00 | 1.624 (1.503, 1.754)* | 1.908 (1.765, 2.064)* | <0.001 |  |
| Multivariable-adjusted OR (95% CI) | 1.00 | 1.272 (1.170, 1.383)* | 1.144 (1.043, 1.255)* | 0.003 |  |
| BMI ≥24 |  |  |  |  |  |
| No. of patients | 6,659 | 9,602 | 10,582 |  |  |
| No. of cases (%) | 1,591 (23.8) | 2,966 (30.8) | 4,135 (39.0) |  |  |
| OR (95% CI) | 1.00 | 1.424 (1.326, 1.529)* | 2.043 (1.908, 2.188)* | <0.001 |  |
| Multivariable-adjusted OR (95% CI) | 1.00 | 1.182 (1.096, 1.276)* | 1.378 (1.274, 1.490)* | <0.001 |  |
| **Hypertension** |  |  |  |  | 0.139 |
| No |  |  |  |  |  |
| No. of patients | 11,858 | 9,832 | 7,818 |  |  |
| No. of cases (%) | 1,699 (14.3) | 2,024 (20.5) | 1,925 (24.6) |  |  |
| OR (95% CI) | 1.00 | 1.550 (1.444, 1.664)* | 1.953 (1.816, 2.101)* | <0.001 |  |
| Multivariable-adjusted OR (95% CI) | 1.00 | 1.178 (1.091, 1.272)* | 1.234 (1.135, 1.342)* | <0.001 |  |
| Yes |  |  |  |  |  |
| No. of patients | 5,145 | 7,147 | 9,178 |  |  |
| No. of cases (%) | 1,423 (27.6) | 2,565 (35.8) | 3,807 (41.4) |  |  |
| OR (95% CI) | 1.00 | 1.464 (1.354, 1.583)* | 1.854 (1.722, 1.996)* | <0.001 |  |
| Multivariable-adjusted OR (95% CI) | 1.00 | 1.226 (1.128, 1.332)* | 1.315 (1.208, 1.431)* | <0.001 |  |
| **Dyslipidaemia** |  |  |  |  | 0.986 |
| No |  |  |  |  |  |
| No. of patients | 12,591 | 10,878 | 10,388 |  |  |
| No. of cases (%) | 1,958 (15.5) | 2,460 (22.6) | 3,013 (29.0) |  |  |
| OR (95% CI) | 1.00 | 1.587 (1.486, 1.695)* | 2.219 (2.081, 2.366)* | <0.001 |  |
| Multivariable-adjusted OR (95% CI) | 1.00 | 1.174 (1.093, 1.261)* | 1.230 (1.140, 1.327)* | <0.001 |  |
| Yes |  |  |  |  |  |
| No. of patients | 4,412 | 6,101 | 6,608 |  |  |
| No. of cases (%) | 1,164 (26.3) | 2,129 (34.8) | 2,719 (41.4) |  |  |
| OR (95% CI) | 1.00 | 1.496 (1.374, 1.629)* | 1.951 (1.796, 2.120)* | <0.001 |  |
| Multivariable-adjusted OR (95% CI) | 1.00 | 1.236 (1.128, 1.354)* | 1.336 (1.215, 1.470)* | <0.001 |  |

*Significant data.

Abbreviations: No., number; BMI, body mass index; eGFR, estimated glomerular filtration rate; OR, odds ratio; CI, confidence interval.

Adjusted for sex, age, education, SBP, SDP, HR, BMI, smoking status, drinking status, HDL, LDL, TG, ALT, AST, eGFR, CVD, antihypertensive drugs, lipid-lowering drugs.

**Table S3.** Sensitivity analysis excludes participants with any missing values (n=48, 406)

|  | Model 1 | | Model 2 | | Model 3 | |
| --- | --- | --- | --- | --- | --- | --- |
|  | OR (95%CI) | *P*-trend | OR (95% CI) | *P*-trend | OR (95% CI) | *P-*trend |
| WWI Tertile |  | <0.001 |  | <0.001 |  | <0.001 |
| T1 | 1.00 |  | 1.00 |  | 1.00 |  |
| T2 | 1.628 (1.595, 1.773)* |  | 1.329 (1.257, 1.406)* |  | 1.227 (1.159, 1.299)* |  |
| T3 | 2.273 (2.159, 2.394)* |  | 1.391 (1.313, 1.473)* |  | 1.291 (1.216, 1.371)* |  |
| WWI Continuous | 1.432 (1.398, 1.466) | <0.001 | 1.145 (1.115, 1.175)* | <0.001 | 1.102 (1.072, 1.132)* | <0.001 |

*Significant data

Model 1: unadjusted;

Model 2: adjusted for sex, age, education, SBP, SDP, HR, BMI, smoking status, drinking status;

Model 3: additionally adjusted for HDL-C, LDL-C, TG, ALT, AST, eGFR, CVD, antihypertensive drugs, lipid-lowering drugs.

**Table S4.** Sensitivity analysis of excluding participants with BMI≥28kg/m^2^ (n=7,233)

|  | Model 1 | | Model 2 | | Model 3 | |
| --- | --- | --- | --- | --- | --- | --- |
|  | OR (95%CI) | *P*-trend | OR (95% CI) | *P*-trend | OR (95% CI) | *P-*trend |
| WWI Tertile |  | <0.001 |  | <0.001 |  | <0.001 |
| T1 | 1.00 |  | 1.00 |  | 1.00 |  |
| T2 | 1.604 (1.517, 1.695)* |  | 1.258 (1.185, 1.334)* |  | 1.184 (1.114, 1.257)* |  |
| T3 | 2.076 (2.965, 2.193)* |  | 1.257 (1.180, 1.340)* |  | 1.187 (1.112, 1.266)* |  |
| WWI Continuous | 1.393 (1.358, 1.430) | <0.001 | 1.088 (1.056, 1.121)* | <0.001 | 1.059 (1.027, 1.092)* | <0.001 |

*Significant data

Model 1: unadjusted;

Model 2: adjusted for sex, age, education, SBP, SDP, HR, BMI, smoking status, drinking status;

Model 3: additionally adjusted for HDL-C, LDL-C, TG, ALT, AST, eGFR, CVD, antihypertensive drugs, lipid-lowering drugs.
